# Community paediatric respiratory infection surveillance study protocol: a feasibility, prospective inception cohort study

**DOI:** 10.1136/bmjopen-2016-013017

**Published:** 2016-08-31

**Authors:** Emma C Anderson, Suzanne Marie Ingle, Peter Muir, Charles Beck, Adam Finn, John Peter Leeming, Christie Cabral, Joanna May Kesten, Alastair D Hay

**Affiliations:** 1 National Institute for Health Research Health Protection Research Unit (NIHR HPRU) on Evaluation of Interventions, University of Bristol, Bristol, UK; 2 School of Social and Community Medicine, University of Bristol, Bristol, UK; 3 Department of Health and Social Sciences, University of the West of England, Bristol, UK; 4 Public Health England, Specialist Virology Centre, Bristol, UK; 5 Field Epidemiology Service, Public Health England, Bristol, UK; 6 Schools of Clinical Sciences and Cellular and Molecular Medicine, University of Bristol, Bristol, UK; 7 North Bristol NHS Trust, Bristol Centre for Antimicrobial Research and Evaluation (BCARE), Bristol, UK; 8 The National Institute for Health Research Collaboration for Leadership in Applied Health Research and Care West (NIHR CLAHRC West) at University Hospitals Bristol NHS Foundation Trust, Bristol, UK

**Keywords:** PRIMARY CARE, PUBLIC HEALTH

## Abstract

**Introduction:**

Paediatric respiratory tract infections (RTIs) are common reasons for primary care consultations and antibiotic prescribing. Locally relevant syndromic and microbiological surveillance information has the potential to improve the care of children with RTIs by normalising illness (parents) and reducing uncertainty (clinicians). Currently, most RTI studies are conducted at the point of healthcare service consultation, leaving the community burden, microbiology, symptom duration and proportion consulting largely unknown. This study seeks to establish the feasibility of (mainly online) participant recruitment and retention, and the acceptability/comparability of parent versus nurse-collected microbiological sampling, to inform the design of a future surveillance intervention study. Evidence regarding consultation rates and symptom duration is also sought.

**Methods and analysis:**

A community-based, feasibility prospective inception cohort study, recruiting children aged ≥3 months and <16 years and their parents via general practitioner surgery invitation letter, aiming to collect data on 300 incident RTIs by July 2016. Following informed consent, parents provide baseline (demographic) data online, and respond to weekly emails to confirm the absence/presence of new RTI symptoms. Once symptomatic, parents provide daily data online (RTI symptoms, school/day-care attendance, time off work, health service use, medication), and a research nurse visits to collect clinical examination data and microbiological (nasal and saliva) swabs. Parents are invited to provide symptomatic (at nurse visit, but without nurse assistance) and asymptomatic (alone) swabs on recovery. A review of primary care medical notes will gather medical history, health service utilisation, referral and antibiotic prescribing rates. Feasibility will be assessed using recruitment and retention rates, data completeness; and acceptability by quantitative survey and qualitative interviews. Symptomatic parent and nurse swab pairs will be compared for microbe isolation.

**Strengths and limitations of this study**

- Near exclusive online methods to recruit and follow-up children with respiratory tract infections not yet brought to the attention of healthcare services.
- Will provide evidence of the feasibility of conducting larger scale community-based studies.
- Will provide novel evidence regarding the natural history of RTI symptoms in a non-consulting paediatric population.
- Will provide novel evidence regarding the proportion of children consulting within any given illness episode.
- Representativeness of study participants may be limited by low response to study invitation.

## Introduction

General practitioners' (GPs) workload is increasing as primary care has seen a rise in consultation rates (without corresponding increase in GP staff time), and increased complexity in patient cases over recent years.^1^ Respiratory tract infections (RTIs) are the most common problem managed by primary care internationally, with the majority occurring in children.^2^ Children experience on average 6–8 RTIs annually and NHS costs and costs to parents and carers (hereafter termed ‘parents’) for paediatric RTIs are significant.^3^

Additionally, there is a growing public health threat of increasing antimicrobial resistance (AMR),^4^ largely attributed to the overprescription of antibiotics.^5^ The use of primary care services for RTIs and antibiotic prescribing are inseparable; primary care is responsible for nearly 80% of antibiotics prescribed^6^ and 54% of patients with RTI are given antibiotics.^7^ Owing to the large numbers, even a small change in consultation rates and improved targeting of antibiotic prescribing for paediatric RTIs could have a significant impact on primary care resources and help reduce AMR.

A key element of the problem is uncertainty felt by parents and clinicians. In parents, uncertainty exists in understanding their child's illness (eg, normal symptoms and duration), appropriate care and when to consult.^7–9^ In clinicians, uncertainty exists regarding diagnosis and effective treatment of RTIs in primary care, indicated by the variation in antibiotic prescribing between clinicians,^10^ and surgeries.^6^ GPs tend towards prescribing antibiotics in the face of uncertainty for paediatric RTIs, due to a perception that not prescribing carries greater potential threat.^11^

The Chief Medical Officer's 2011 Annual Report identifies a need for enhanced surveillance of infectious diseases, with a focus on tackling AMR and improving antibiotic stewardship, and recommends research to enhance infectious disease surveillance.^12^

Currently available UK healthcare-based infection surveillance information includes microbiology data presented in weekly laboratory reports from the Public Health England (PHE) based on swabs predominantly received from secondary care. These data are not currently routinely provided to GPs as an informational resource. Syndromic surveillance is also underway via PHE weekly summaries of broad syndromic illnesses presenting to healthcare services (primary and secondary care). There is an absence of matched microbiology and syndromic data, and an absence of community infection data of any kind, much less community paediatric RTI data. Enhanced surveillance can provide these data, which could better inform clinical judgement when dealing with this highly prevalent patient population.

Community participation in illness surveillance (via real-time online symptoms self-report) has been successfully applied to influenza in adults—for example, Gripenet^13^ and the FluSurvey project,^14^ though the majority of this work to date has been based on symptom self-report, and lacks associated microbiological data to identify circulating pathogens.

Improving the availability of surveillance data on community RTIs, including the microbiology matched with syndromic presentation, has the potential to reduce clinician uncertainty and improve the targeted use of antibiotics and appropriate secondary care referrals. It may also improve clinicians' ability to reassure patients, specifically since parents find that commonly used microbiological diagnoses (eg, ‘it's just a virus’) in the absence of microbiological evidence undermine clinician credibility.^7^ The availability to parents of community infection surveillance data is hypothesised to have potential to decrease parental anxiety and encourage home management of RTIs, thereby reducing consultation burden.

Additionally, previous research has focused on paediatric RTIs captured during primary care presentation.^15^ ^16^ This leaves a key area of uncertainty regarding community paediatric RTIs. Duration, symptoms and proportion seen in primary care are not known, nor is the relative contribution of socio-demographic, clinical and microbiological factors to primary care consultation for RTIs. Our study will be able to provide some answers to these questions by collecting prospective data on paediatric RTIs in the community as well as presenting a comparison of the duration of RTI symptoms between consulting and non-consulting children, which has potential for informing other interventions (beyond the planned surveillance intervention to be built from this study).

### Aim

The primary aim is to assess the feasibility of assembling a non-consulting inception cohort from whom paediatric RTI microbiological and symptomatic surveillance data are collected. The purpose is to inform the design of future surveillance studies and interventions, with the overarching objective to improve primary care utilisation, and antibiotic prescribing for paediatric RTI. Secondary aims are to provide evidence regarding the proportion choosing to consult for reported symptoms and the duration of RTI symptoms in the community.

## Methods and analysis

### Sample selection

#### GP surgery recruitment

**Figure 1:**
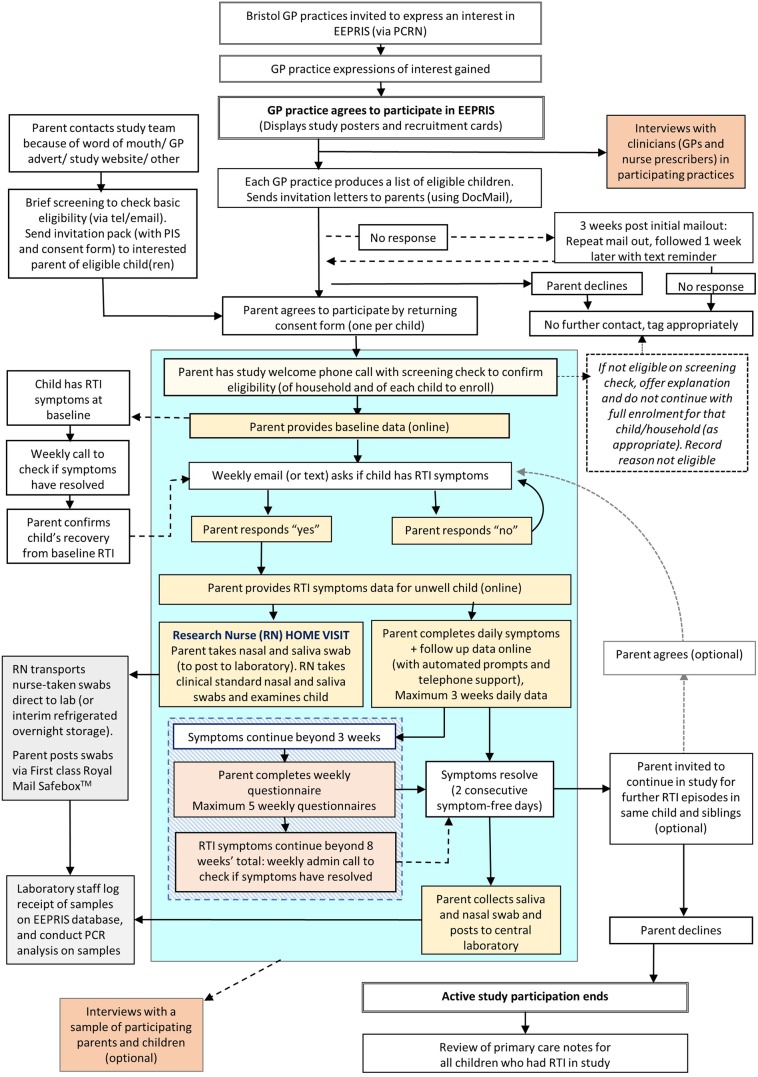
Study recruitment and data collection flow chart. Blue box indicates main study processes for enrolled participants, yellow boxes indicate participant actions to take, grey boxes indicate laboratory processing and orange boxes indicate interviews (optional).

An invitation was sent to all GP surgeries within a 10 mile radius of Bristol City Centre via the NIHR Clinical Research Network (CRN), West of England. Nineteen surgeries expressed interest, of which 10 reached and confirmed agreement with the study team to take part (figure 1). Efforts were made to source GP surgeries across a range of areas of deprivation.

#### Participant screening and exclusion

GP practices were asked to run database searches with filters to exclude: children aged under 3 months or over 15 years; children with life-limiting (terminal) illness (for whom a study invitation could cause distress); those with a health condition giving greater risk of serious infection; and temporary (as opposed to fully registered) patients. GP surgery staff further screened lists to remove: children resident in households for which a study invitation could cause stress to the parents (eg, recent bereavement or terminal illness in the family); or which may present a risk for a research nurse (RN) or interviewer conducting a lone home visit (eg, history of domestic violence).

The process for letter invitation to take part involved uploading the final patient list to an online mailing company, which sent an invitation letter addressed ‘to the parent/carer of [name of child]’ together with an information leaflet, consent form and FREEPOST reply envelope addressed to the study team. All children aged 7 or older by September 2015 (the equivalent of UK school year 3 and above) also received a children's information leaflet, and assent form to sign.

Parents return signed consent (and assent) forms to the study team using pre-paid envelopes. On receipt of consent, the study team confirm eligibility over the telephone to enrol children into the study and initiate online data collection. In addition to the exclusion criteria above, children are excluded if: the parent lacks capacity to consent; or lacks adequate English language understanding to complete study processes or other reasons (eg, family away during data collection phase).

#### Supplementary recruitment efforts

Attempts were made to maximise response rates through supplementing the initial letter mailout with non-responders receiving a reminder letter and (where the GP surgery had the facility) text (texts were not sent to the mobile numbers associated with older children's medical records due to the risk of the number being the child's own phone rather than the parents, who are the consenting participants). In addition, posters and recruitment cards were displayed in all GP surgeries; the team released a press release and set up a study website encouraging recruitment. Recruitment cards were also sent to all families identified as eligible during their welcome call, with a request to pass the message on to other parents who may be interested in the study (for ‘snowball’ recruitment).

#### Families, children and incident RTIs

The event of interest are new RTIs, developing after study recruitment. Participants are children registered at participating GP surgeries, while parents are the main point of contact for study participation, since they provide consent and data for the participating children. Children aged 7 by September 2015 (equivalent to UK school year 3) or older provide assent prior to participation. A subsample of participating parents and children will be interviewed, as well as a subsample of clinicians from participating GP surgeries.

GP surgeries provide basic, anonymised information regarding non-responders (gender and age of children) and a measure of index of multiple deprivation (IMD) for comparison with the recruited cohort. Recruiting via GP surgeries therefore brings the benefit of enabling some assessment of how representative the recruited cohort is for each surgery.

### Sample size

**Figure 2:**
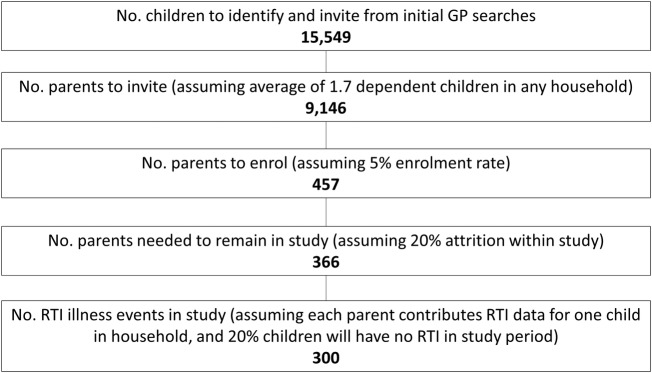
Study recruitment and retention projection.

The study is set up to provide descriptive results to inform the design of future community surveillance and intervention studies. In this context, recruitment and retention are the primary outcomes of this feasibility study. Incident RTIs developing before mid-July 2016 is the unit of analysis, defined as parent-reported new RTI symptoms (blocked/runny nose, earache/ear discharge, sore throat, cough or chesty symptoms—over and above normal). A sample size of 300 incident RTIs is set as an aim, as a potentially attainable number based on practical considerations (figure 2).

In addition to retention and recruitment rates, the study will address two main secondary aims, with statistical calculations outlined below:

1. How many children with RTI consult?
2. What is the duration of the RTI symptoms in the community?
3. Consultation rate: Approximately 20% of adults with RTI in the community consult with a clinician,^17^ while equivalent rates in children are unknown (a research gap that this study addresses). With a sample size of 300 RTI, we could expect a 95% CI of ±5% (15% to 25%) around a 20% point estimate for consultation rates.
4. Duration of symptoms: We could expect a 95% CI of 87% to 93% around an estimate of 90% for the proportion recovered at the relevant time point, using an exact binomial calculation (using the same methods used for Hay *et al*'s^18^ study on cough duration).

Figure 2 shows a flow chart of projected recruitment numbers based on conservative estimates of 80% of recruited children developing at least one RTI over the study period,^2^ 20% parent attrition rate after study enrolment and that each parent will contribute data for just one episode in one child only rather than opting to continue in the study beyond this. By these calculations, we would need to recruit a total of 457 parents to take part in the study, or 777 children (based on an average 1.7 dependent children per UK household).^19^ Based on previous similar research by the wider group, mailout response rate was expected to be 5%.

### Outcomes

#### Primary outcome

The primary outcome is recruitment and retention. Recruitment rates are the number of incident RTIs reported in the study (and the proportion of these per total recruited children/families). Retention is measured as the number of incident RTI episodes in the study with a reported recovery date (defined as two consecutive symptom-free days), to be presented with a descriptive analysis of missing data

The main secondary outcomes are:

1. Primary and secondary care consultation rates as a proportion of reported episodes of RTI symptoms.
2. Duration of RTI symptoms, including a comparison between consulting and non-consulting children.

Further secondary outcomes:

1. RTI numbers contributed per child and per household (ie, including enrolled siblings).
2. Subassessment of rates of retention in the study (eg, agreeing to ongoing symptoms trigger texts/emails) after completion of one RTI episode data; response rates to weekly emails.
3. Representativeness of study sample via comparison of responders with non-responders to GP surgery invitation letter with respect to family deprivation level assessed via IMD (derived from parent address postcode and GP practice population, respectively).
4. Completion rates of survey questions including: use of primary and secondary care services for RTIs prior to RTI symptom resolution; prescribing and consumption of antibiotics; parental health anxiety; parent confidence in managing children's minor illness; adverse events (AEs); costs to the NHS (eg, cost of consultations and prescriptions) and the family (eg, over the counter medicines) and consequences (eg, time off school/nursery, time off work), with preliminary statistical analysis of these data.
5. Factors associated with decisions to consult (measured directly via free-text response to the online survey question ‘what triggered that decision’, elicited on parent report of consulting for incident RTI; together with a descriptive analysis of other factors including parent-reported health-anxiety and household demographics).
6. Cost of feasibility study with scaled up cost estimate of a definitive cohort study with real-time online syndromic/microbiological surveillance intervention.
7. Acceptability of study processes (eg, online surveys and swab sampling), collected via parent and children interviews and supplemented by parent survey (asking for 5-point Likert scale response to how user-friendly parents found the online questionnaire; ‘very’ to ‘not at all’ with optional free text box).
8. Attitudes towards the informational content, design and perceived potential impact of infection surveillance information, collected via parent and clinician interviews (based on presentation of mock-up example data) to inform intervention design and development.
9. Agreement of microbiological detection between:
  - Nasal swabs versus saliva samples (as κ statistic);
  - Parent-collected and posted nasal swabs compared with ‘gold standard’ RN-collected nasal swabs (as κ statistic);
  - Parent-collected and posted saliva samples compared with ‘gold standard’ RN-collected saliva samples (as κ statistic).
10. Difference in microbiological presence and load (cycle threshold values) between symptomatic and asymptomatic samples as a matched comparison with each RTI case, using paired t-tests/Wilcoxon signed-rank tests.

### Data collection

Figure 1 shows the study recruitment and data collection flow chart, with details of the data to be collected outlined below. Parent-reported data are collected using an online database, designed and built specifically for the study, which provides survey links and reminders to parents via automated emails.

1. *Baseline data* (collected online prior to first study RTI): Parent, child and household demographics (including ethnicity of parent and child, parent employment status and educational level, ages of members of the household) and optional parent health anxiety/confidence scales.
2. *Incident RTIs* (<7 days of symptom onset): Parents are emailed weekly and asked to confirm (simple Y/N response) if each child has developed: blocked/runny nose, earache/ear discharge, sore throat, cough or chesty symptoms in the last 7 days over and above what is normal for them. A ‘Y’ response elicits a symptoms and recovery survey for the parent to complete online (via smartphone, tablet or computer) to record symptom start date and ongoing daily symptoms.
3. *Microbiological sampling*: A ‘Y’ response also prompts the RN to arrange a home visit while the child is symptomatic for clinical examination of the child and to collects a set of saliva and nasal swabs from the child, transported via preservative broth in collection tubes immediately to the laboratory (‘gold standard’ sampling). The RN also asks the parent to take a saliva and nasal swab from the child during the visit, using dry collection tubes and post these to the laboratory. Laboratory analysis of swab samples uses PCR on an array of common respiratory tract viruses and bacteria.
4. *Follow-up RTI data*: Parents are asked to continue to complete an online symptoms diary to record: RTI symptoms and severity until illness resolution (two consecutive RTI symptom-free days), and impact of the illness (school/day-care attendance; time off work; symptomatic status of household members, primary care consultation, antibiotic consumption and other medication use). Daily symptoms data are collected for a maximum of 21 days. If symptoms are ongoing beyond 21 days, data collection reverts to a weekly online questionnaire up to a maximum of 8 weeks from the date of first RTI symptom presentation. Following symptom resolution, the parent can choose to opt in or out of continuing to participate in the study with the same child or any other children in the household (ie, resume receiving weekly emails in the case of further RTI development in a child). Reminders for diary completion are via an automated email sent every 2 days. Complementary telephone calls encourage survey completion if missing data lags for more than a week.
5. *Asymptomatic sample*: As soon as possible after symptom resolution, the parent is asked to collect a saliva and a nasal swab (without RN visit) to post to the laboratory for PCR analysis as a matched case comparison, and to complete a final online swabs collection survey (to confirm presence, if any, of symptoms at the time of swab collection, date of collection or reason for non-collection of samples).
6. *Follow-up primary care notes data*: will record primary care RTI consultations and antibiotic prescribing during the RTI, as well as secondary care referrals or contacts, and diagnostic information relating to RTI (conducted on all children contributing RTI data to the study; collected at the end of the ‘live’ data collection period). Relevant medical history will also be extracted to supplement baseline data.
7. *Parent (and child) interviews* (planned n=30): Of the parents who (on study enrolment) consented to an interview, a maximum diversity sample will be invited for one face-to-face semistructured interview (at parent's home or convenient location). These interviews aim to understand parent and child experiences and acceptability of the study components as well as gaining feedback on the content and utility of the planned intervention.
8. *Clinician interviews* (planned n=24): Clinicians in participating GP practices will be invited for 130 min semistructured interview (in person/via telephone) to gain feedback on the acceptability and potential impact of the planned intervention.

## Data analysis plan

As this is a feasibility study, we will present simple descriptive results. The main outcome, recruitment and retention rates, will be presented with a descriptive analysis of missing data.

We will describe the characteristics (to include age, comorbidities and ethnicity) of the children and households in the study (means and SDs will be presented for normally distributed variables, medians and IQRs will be presented where variables are non-normally distributed, and percentages presented for categorical variables). Descriptive characteristics of similar variables (as available from GP database search outputs) will be presented for the GP surgery populations from which we recruited (for sample representativeness and response rates).

### Analysis of primary outcome

We will assess whether our outcome (recruitment and retention) is associated with particular baseline characteristics, for example, does response completion vary by postcode index of deprivation or household crowding? Is age, gender or ethnicity of the child or parent related to response rate? As our primary outcome is represented by binary variables: we will use χ^2^ tests, t-tests and univariable logistic regression to estimate associations of baseline characteristics with outcome. Regression analyses will be adjusted for clustering (children within families within GPs) where appropriate.

### Analysis of secondary outcomes

Similar strategies will be used for the secondary outcomes and linear regression will be used where the outcome is continuous, for example, duration of RTI symptoms. Again, regression analyses will be adjusted for clustering where appropriate.

We will use κ statistics to assess the level of agreement between different methods of collecting swabs. If we consider the RN-collected swabs to be the gold standard, then we can assess the diagnostic utility of the parent-collected swabs by calculating sensitivity and specificity. We will also consider whether the quantity of microorganisms present differs according to the collection site (nasal or mouth) by calculating correlation coefficients and limits of agreement for the Bland-Altman plots.

All statistical analyses will be completed using Stata V.14 (StataCorp. Stata Statistical Software: College Station, Texas: StataCorp LP, 2015).

Interview data will be audio recorded using encrypted devices, transcribed verbatim and analysed via NVivo V.10 using the framework method.^20^

## Ethics and dissemination

### Ethical considerations

The study is low risk in terms of ethical issues. Children will not be receiving a medication or intervention, and no treatment is withheld. The cost to parents is mainly in the time taken to fill in questionnaires and a potential ‘burden’ of reading and responding to regular emails. A patient and public involvement (PPI) group of Bristol parents were consulted several times during the study design to ensure the paperwork, surveys and procedures were likely to be acceptable. Participants who develop an RTI, complete symptom diaries and provide swab samples will receive a £15 high street voucher as a thank you gift for their time. Parents who take part in the interview study will receive an extra £5 voucher.

#### Patient safety

Patient safety takes highest priority throughout all study procedures.

Nasal swab-taking by parents may cause minor distress either to parents or to the child, but are not likely to cause physical harm. Saliva sample-taking is an unobtrusive process with very low likelihood of causing any distress. The study team provides clear instructions and support (if necessary) for parents on swab-taking. The RN procedures (taking saliva and nasal swab and a clinical examination) are routine procedures, undertaken by experienced professionals. In the event of significant child or parent distress around the process, no swabs or examination are to be taken. RNs act according to clinical expertise and NHS guidelines when conducting visits to prioritise patient safety. In the event that the RN, on assessing the child, is concerned about the health of the child, they may advise that the parent seeks medical advice (recording this as a possible study-induced primary care visit).

Participants retain the right to withdraw from the study at any time, with an additional right to request destruction of existing data or samples collected from them.

#### Research staff safety

Home visits conducted by RN and qualitative interview staff are guided by the University of Bristol safety policy for researchers working in the field.^21^ A lone worker policy is applied for all fieldwork in accordance with University of Bristol guidance.^22^

#### Study conduct

RNs and all the research team are DBS checked for participant safety measures (according to the principles of GCP).

Medical notes review will be undertaken by research staff with honorary NHS contracts and the process will be conducted according to NHS and research standards on confidentiality and ethical practice.

### Safety (AEs)

Any unexpected AEs defined as ‘any untoward medical occurrence in a study participant’ and serious adverse events (SAEs) will be monitored by the study team for seriousness, causality and expectedness, and these will reviewed at management group meetings.

An SAE is defined as any untoward and unexpected medical occurrence or effect that: results in death; is life-threatening (refers to an event during which the participant was at risk of death at the time of the event; it does not refer to an event which might have caused death had it been more severe in nature), requires hospitalisation, or prolongation of existing hospitalisation; results in persistent/significant disability or incapacity; is a congenital abnormality or birth defect.

#### AE procedure

In the event that a research team member is informed of a hospitalisation or other SAE occurring within the active participation phase of the study, the research team member will record relevant information about the incident, including whether the study was in any way linked to the outcome. The research team member initially collecting this information will report the incident to the PI and/or study manager within 24 hours of its discovery (in accordance with usual SAE procedures). If there is any indication that an SAE could have been linked to study procedures (eg, a child inhaling a saliva sample), it will provoke a research team-led investigation, with reporting to the sponsor via the usual procedures as detailed on the University of Bristol Research and Enterprise Development website.^23^

Around 1% of children presenting to primary care with RTI are likely to be hospitalised.^24^ With a sample of 300 RTI episodes, a generous allowance of up to one-third of children with RTI presenting to primary care would anticipate that just one child may be hospitalised for their RTI within the study duration.

All expected SAEs will be reported as part of the outcome of the study.

### Dissemination plan

The written papers from this work will be submitted for publication in quality peer-reviewed health journals. There will be a main feasibility study paper which will inform the design of future cohort and intervention studies. Owing to their novelty, we anticipate that secondary study results will be publishable as separate papers, for example, the clinical utility and cost of community swab types; RTI duration in the community and numbers of children consulting; microbiological (and symptoms) profiling of RTIs (with possible future transmission modelling); contribution of socio-demographic, clinical and microbiological factors to NHS use; qualitative papers describing parent and clinician views of a microbiological information intervention. Application will be made to present study results at relevant conferences.

Study participants will receive a newsletter outlining the main study results. The PPI group will advise on parent newsletter content and dissemination. Participating GPs will also receive a newsletter about the results. These newsletters will include a link to the study website where we will post further information and links to papers when accepted for publication.
